# Lead exposure study among workers in lead acid battery repair units of transport service enterprises, Addis Ababa, Ethiopia: a cross-sectional study

**DOI:** 10.1186/1745-6673-3-30

**Published:** 2008-11-28

**Authors:** Kemal Ahmed, Gonfa Ayana, Ephrem Engidawork

**Affiliations:** 1 Department of Pharmacology, School of Pharmacy, Addis Ababa University, Addis Ababa, Ethiopia; 2 Department of Clinical Chemistry, Ethiopian Health and Nutrition Research Institute, Addis Ababa, Ethiopia

## Abstract

**Background:**

Lead exposure is common in automobile battery manufacture and repair, radiator repair, secondary smelters and welding units. Urinary Aminolevulinic acid has validity as a surrogate measure of blood lead level among workers occupationally exposed to lead. This study had therefore assessed the magnitude of lead exposure in battery repair workers of three transport service enterprises.

**Methods:**

To this effect, a cross-sectional study was carried out on lead exposure among storage battery repair workers between November 2004 and May 2005 from Anbasa, Comet and Walia transport service enterprises, Addis Ababa, Ethiopia. Subjective information from the workers was obtained by making use of structured questionnaire. Other information was obtained from walkthrough evaluation of the repair units. Aminolevulinic acid levels in urine were used as an index of the exposure. This was coupled to measurements of other relevant parameters in blood and urine collected from adult subjects working in the repair units as well as age matched control subjects that were not occupationally exposed to lead. Aminolevulinic acid was determined by spectrophotometry, while creatinine clearance, serum creatinine, urea and uric acid levels were determined using AMS Autolab analyzer.

**Results:**

Urinary aminolevulinic acid levels were found to be significantly higher in exposed group (16 μg/ml ± 2.0) compared to the non-exposed ones (7 μg/ml ± 1.0) (p < 0.001). Alcohol taking exposed subjects exhibited a significant increase in urinary aminolevulinic acid levels than non-alcohol taking ones (p < 0.05). Moreover, urinary aminolevulinic acid levels of exposed subjects increased with age (p < 0.001) as well as duration of employment (p < 0.001). Whereas serum uric acid levels of exposed subjects was significantly higher than non-exposed ones (p < 0.05), no statistically significant difference had been found in renal indices and other measured parameters between exposed and non-exposed subjects. From the questionnaire responses and walkthrough observations, it was also known that all the repair units did not implement effective preventive and control measures for workplace lead exposure.

**Conclusion:**

Taken together, these findings indicated that workers in lead acid battery repair units of the transport service enterprises are not protected from possibly high lead exposure. Thus, strict enforcement of appropriate and cost effective preventive and control measures is required by all the enterprises.

## Background

Lead (Pb) is a highly toxic metal with no known physiological benefits and is a ubiquitous pollutant in the ecosystem as a result of its natural occurrence and its industrial use. Mankind has used lead for over 6000 years [1]. Lead's toxicity was recognized and recorded as early as 2000 BC and the widespread use of lead has been a cause of endemic chronic plumbism in several societies throughout history.

Significant occupational lead exposures are not limited to traditional heavy industries. Automobile battery manufacture and repair, radiator repair, secondary smelters (including scrap metal refiners) are found in most countries and are common sources of lead exposure. These small domestic versions of secondary smelters are typically located within or in close proximity to homes and lead fumes and dust generated in such operations also poses health hazard to children and adults [1,2]. In developing countries the distinction between home and workplace lead exposure is non-existent [3].

The prevention of occupational hazards is far more effective and less costly when considered during the early stages. Lead poisoning amongst occupationally exposed persons is known to pose serious health problems on the nervous system, heme biosynthesis, kidneys, reproductive system, hepatic, hearing, endocrinal, gastrointestinal, blood pressure and cardiovascular system [4-6]. The effect of lead on heme synthesis is attributed to inhibition of enzymes involved in heme synthesis, resulting in abnormal concentrations of heme precursors in blood and urine. Essentially, lead interferes with the activity of three enzymes: it indirectly stimulates the mitochondrial enzyme aminolevulinic acid synthetase (ALAS); directly inhibits the activity of the cytoplasmic enzyme aminolevulinic acid dehydratase (ALAD); and it interferes with the normal functioning of intramitochondrial ferrochelatase [2]. The functional changes on kidney are related to lead effect on mitochondrial respiration and phosphorylation in proximal tubules of nephron [5]. Typical measures of renal failure, e.g. blood urea nitrogen (BUN) and creatinine are elevated as a consequence of lead induced renal failure. Chronic occupational lead exposure is also related to low urate excretion and a high incidence of gout in lead workers [7].

Significant human suffering related to occupation is unacceptable and often results in appreciable financial loss due to the burden on health and social security systems, which negatively impacts production [8]. There are a number of occupational hazards in all workplaces worldwide due to lack of adequate prevention and control measures [9]. Occupational exposure to lead still occurs in many countries of the world. Especially in many developing countries, occupational lead exposure is entirely unregulated and no monitoring of exposures exists [10]. The present study was therefore aimed at investigating lead exposure among lead-acid battery repair workers and relating the exposure to health effects.

## Methods

### Study population

A total of 51 subjects (45 male and 6 female) aged between 23 and 57 years and who had worked for over six months in lead acid battery repair units of transport service enterprises in Addis Ababa (Anbasa, Comet and Walia) had participated in this study (Table 1). Fifty healthy non-exposed age matched subjects (48 male and 2 female) were taken as control for comparison with exposed group.

**Table 1:** Study sites and demographic data of lead exposed workers (n = 51)

**Exposed workers**	**Number**	**%**
**Enterprise**		
**Anbasa**	23	45.1
**Comet**	17	33.3
**Walia**	11	21.6
**Age**		
**20–35**	4	7.9
**36–45**	22	43.1
**46+**	25	49.0
**Sex**		
**Male**	45	88.2
**Female**	6	11.8

All subjects were informed about the purpose, benefits and risks of the study and their right to withdraw at any time point. Following this, each of them had given their consent of participation in the study. The study was approved by the IRB of the School of Pharmacy, Addis Ababa University. For each subject, information on personal particulars, work experience, health risks and other relevant factors that might influence lead exposure were collected using a pre-tested and standardized structured questionnaire.

### Sample collection

Urine samples were collected between 9:00 and 11:00 a.m. from study participants using light protected plastic urine containers (wrapped with aluminum foil), which contained 2 g barbituric acid as preservative. The measurement of volumes was done using graduated cylinder. In parallel, 3 ml blood samples were collected and centrifuged, and serum was separated for analyses. Both serum and urine specimens were refrigerated at 4°C immediately in the dark until time of analyses. The specimen container was labeled with codes that represent each participant; date and time of collection for identification purposes [11,12].

### Measurement of urinary delta-Aminolevulinic acid

Urinary delta-Aminolevulinic acid (δ-ALA) levels were determined spectrophotometrically as described elsewhere [13]. Briefly, urine samples were heated with buffered ethyl acetoacetate to produce pyrrole derivatives. This δ-ALA derivative was purified by extraction into ethyl acetate. Ehrlich reagent was then added to produce a reddish color and absorbance was measured at 553 nm. For the analyses, four tubes were prepared as follows: Tube A: Water blank (1 ml water +1 ml acetate buffer + 0.2 ml ethyl acetoacetate + 3 ml ethylacetate + 2 ml Ehrlich's reagent), Tube B: Subject specimen blank (1 ml urine + 1 ml acetate buffer + 3 ml ethylacetate + 2 ml Ehrlich's reagent), Tube C: Subject specimen (1 ml urine + 1 ml acetate buffer + 0.2 ml ethyl acetoacetate + 3 ml ethylacetate +2 ml Ehrlich's reagent), and Tube D: Subject specimen (1 ml urine + 1 ml acetate buffer + 0.2 ml ethyl acetoacetate + 3 ml ethylacetate + 2 ml Ehrlich's reagent). Tube A served as a blank for tube B while tube B was a blank for tubes C and D. All tubes were heated in a boiling water bath for 10 min and allowed to cool in cold water. The glass stoppers were then removed and centrifuged (1000 g ×) for 1 min to separate the phases. 2 ml of the upper ethyl acetate phase was removed using volumetric pipette. Then, Ehrlich's reagent was added, mixed, left for 10 min and the absorbance at 553 nm was taken using water to zero the spectrophotometer.

For calibration known concentrations of δ-ALA in μg/ml were analyzed by regression analysis to establish the best line that relates measured absorbance to concentration. The analysis gave the following least squares equation, which was used to calculate δ-ALA concentration in μg/ml.

X = Y - 0.01953/0.06399, where X = concentration of δ-ALA (μg/ml) and Y = absorbance.

The urinary levels of δ-ALA were then categorized into four groups, i.e. normal range (<6 μg/ml), acceptable (6–20 μg/ml), high (20–40 μg/ml) and dangerous (> 40 μg/ml) [14].

### Measurement of creatinine clearance, urea and uric acid

Creatinine clearance was used to assess glomerular filtration rate (GFR) function after determining the serum and urinary creatinine concentrations and urine volume over 2 h. Fluitest kit (Biocon^® ^Diagnostic Hecke 8, 34516 Vöhl/Marienhagen, Germany) based on Jaffe Kinetic Colorimetric Method was used for the determination of creatinine. Measurements were done using AMS Autolab analyzer (Roche, Basel, Switzerland). Urea Kit (Biocon^® ^Diagnostic Hecke 8, 34516 Vöhl/Marienhagen, Germany) based on Berthelet method was used for the determination of urea, whilst uric Acid was analyzed using uric Acid PAP Kit (Human Biological Diagnostic, Germany). These tests were also run on the same AMS Autolab analyzer described above.

### Statistical analyses

Data were entered using Excel spread sheet and results were analyzed using STAT ver 6. Student t-test was used to compare urinary δ-ALA level per se and in relation to sex and alcohol taking habits as well as blood uric acid, serum creatinine, creatinine clearance and serum urea levels of both exposed and non-exposed groups. F-ANOVA was also done to relate levels of δ-ALA with duration of exposure, age and enterprises. Values are expressed as means ± SEM. A probability value of less than 5 percent was used as the level of significance. The distribution was regarded as normal distribution and reference values were also considered.

## Results

The distribution of exposed subjects was Anbasa (45.1%), Comet (33.3%) and Walia (21.6%) and about 88% of the interviewed study participants were males (Table 1). The duration of employment in the same position ranged from 1 up to 32 years (Table 2).

**Table 2:** Employment duration of lead exposed workers.

**Employment duration (years)**	**Number of workers**	**%**
≤ 10	9	17.7
11–20	17	33.3
21–25	9	17.7
25+	16	31.3

### Levels of urinary δ-ALA

**Figure 1:**
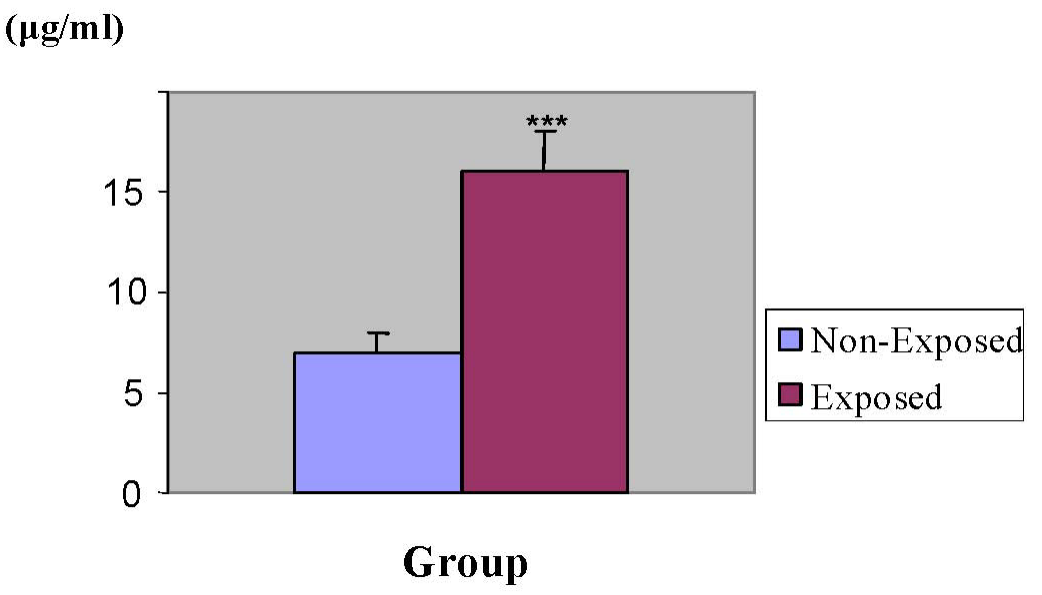
**Urinary ALA levels in exposed and unexposed subjects**. urine samples collected from 51 exposed and 50 non-exposed persons were analyzed for levels of δ-ALA using double beam spectrophotometer. Inter-group analysis was performed using Student t-test. ***P < 0.001.

**Figure 2:**
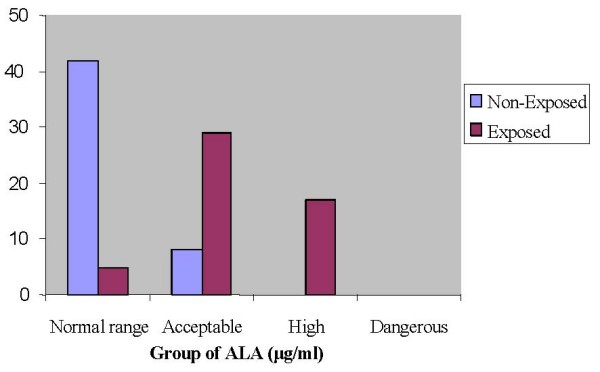
**Distribution of subjects by group using urinary ALA levels**. urinary ALA levels determined as described in the legend of Fig. 1 were used to classify subjects into different groups using ranges given by the manufacturer of the kit i.e. normal (<6 μg/ml), acceptable (6–20 μg/ml), high (20–40 μg/ml) and dangerous (>40 μg/ml).

Biochemical analysis of urinary δ-ALA revealed a two-fold increase (p < 0.001) in exposed than non-exposed subjects (Fig. 1). The mean levels of urinary δ-ALA were 16 ± 2.0 μg/ml in exposed subjects and 7 ± 1.0 μg/ml in non-exposed ones. Exposed and non-exposed subjects were categorized using classification proposed [14] to see the intra-group distribution of δ-ALA. Accordingly, whilst 84% of subjects from the non-exposed group were within normal range, the percent for exposed ones was as low as 9.8% (Fig. 2). Furthermore, more than half of the exposed subjects had acceptable levels and about a third had high levels. By contrast, among non-exposed subjects about 16% displayed acceptable range and none of them had high levels of urinary δ-ALA.

Inter-enterprise analysis of urinary δ-ALA was also done to have an idea whether preventive measures were in place or not. Although levels in Comet (12.6 ± 2.9 μg/ml) tended to be lower than the other two (18.3 ± 3.9 μg/ml for Anbessa and 18.7 ± 6.0 μg/ml for Walia), it failed to reach statistical significance. Categorization of exposed subjects using Lane et al's classification was also applied to enterprises and no dangerous levels had been found in any of the Enterprises. However, the rank order of proportion for high urinary δ-ALA levels was Anbessa ≥ Walia>>>>Comet, with the proportion being about 50% for the former two and about 6% for Comet.

To examine whether urinary δ-ALA levels vary with age, subjects were stratified into different age groups and statistical analysis was performed. The result indicated that urinary δ-ALA levels increased with age in exposed group (p < 0.001) but failed to show any significant difference in non-exposed group (Table 3). Likewise, analysis made to assess the impact of sex on urinary δ-ALA levels failed to show any significant sex-related differences, although levels in male (16.9 ± 2.6 μg/ml) tended to increase than females (13.4 ± 4.5 μg/ml).

**Table 3:** Levels of urinary δ-ALA by age group

**Age**	**δ-ALA (μg/ml) ± SEM**	
	
	**Non-Exposed**	**Exposed**
20–35	7.3 ± 2.2	6.1 ± 2.2***
36–45	6.7 ± 1.2	12.4 ± 3.2***
46+	6.8 ± 1.4	21.7 ± 2.5***

Urinary ALA levels determined as described in the legend of Fig. 1 were compared between similar age groups of exposed and non-exposed subjects. Data were analyzed using F-ANOVA. ***P < 0.001.

The impact of duration of employment on levels of urinary δ-ALA was also analyzed and δ-ALA was found to be a function of duration of employment (Table 4). Indeed, δ-ALA was noted to significantly increase with duration of employment (p < 0.001).

**Table 4:** Urinary δ-ALA (μg/ml) mean levels of exposed workers by employment duration.

**Employment duration**	**δ-ALA (μg/ml) ± SEM**
≤ 10	5.4 ±1.6
11–20	14.5 ± 3.1
21–25	19.0 ± 3.7
25+	23.4 ± 3.5

### Serum creatinine, creatinine clearance and urea levels

In order to see the long-term effects of lead on kidney, different renal indices were measured in both exposed and non-exposed groups and the results are presented in Table 5. No detectable differences were observed in serum creatinine, creatinine clearance and blood urea levels between exposed and non-exposed groups. However, it is worth noting that creatinine clearance decreased by about 11% in exposed subjects, although it fell short of reaching statistical significance. In parallel, an attempt was made to look whether there was deviation from reference values given by the manufacturer and interestingly all were found to lie within the normal range. The normal ranges according to the manufacturer of the kit were: serum creatinine (male, 7–13 μg/ml and female, 6–11 μg/ml); creatinine clearance (male, 94–140 ml/min and female, 72–110 ml/min); and blood urea (150–450 μg/ml for both sex).

**Table 5:** Serum creatinine, creatinine clearance and urea levels of exposed and non-exposed groups.

**Group**	**Serum creatinine (μg/ml) ± SEM**	**Creatinine clearance (ml/min.) ± SEM**	**Urea (μg/ml) ± SEM**
**Non-Exposed**	11.5 ± 0.3	115.33 ± 5.20	218.0 ± 7.9
**Exposed**	11.8 ± 0.2	102.91 ± 6.27	229.1 ± 7.9

### Uric acid levels

**Figure 3:**
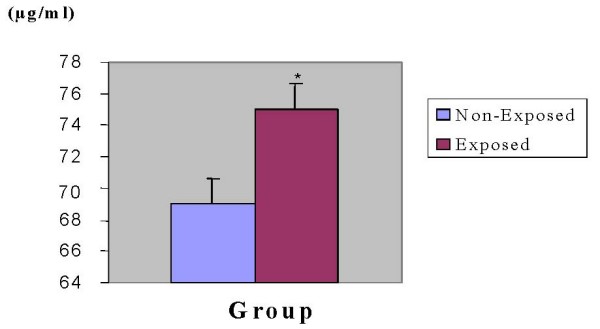
**Levels of blood uric acid levels in exposed and non-exposed subjects**. blood samples were taken from 51 exposed and 50 non-exposed persons and compared for levels of uric Acid using AMS Autolab analyzer. Inter-group variation was analyzed using Student t-test. *P < 0.05.

Lead is known to inhibit uric acid secretion thereby increasing serum uric acid levels. Serum uric acid levels were therefore measured to use it as an indirect measure of lead exposure, along with urinary δ-ALA. Consistent with the aforementioned notion, exposed subjects displayed increased uric acid levels than non-exposed subjects, which were significantly higher by about 8% in exposed subjects than non-exposed ones (p < 0.05) (Fig. 3).

**Figure 4:**
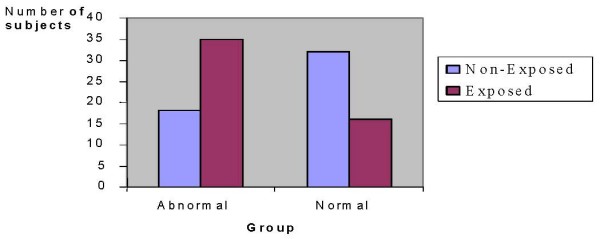
**Distribution of subjects by group using blood uric acid levels**. uric acid levels determined as depicted in the legend of Fig. 3 were used to stratify subjects into different groups using ranges given by the manufacturer of the kit.

Intra-group sub-classification of uric acid levels using normal ranges supplied along with the kit revealed that about 69% exposed subjects had abnormal serum uric acid levels, while this was 36% in non-exposed subjects (Fig. 4). Uric acid normal range was 34–70 μg/ml in male and 26–60 μg/ml in female.

### Data mined from questionnaire

Reported illnesses that were compiled from the structured questionnaire included illnesses linked with lead poisoning, while life style factors were alcohol intake, smoking, meals at workplace and work related hobbies which could result in additional exposure to lead. Twenty of the exposed workers interviewed during this study reported that they had suffered from illnesses, which are known to be commonly linked with lead poisoning and include, among others, visual problems, asthma, gastrointestinal and kidney problems (in order of proportion of respondents).

The effort to associate the habit of alcohol drinking and lead exposure revealed that 60.8% of the respondents were alcohol takers, consuming approximately 6 glass of draught beer per week. Levels of δ-ALA were found to be significantly higher (p < 0.05) in alcohol taking workers (18.9 μg/ml ± 1.5, n = 31) than non-alcohol taking ones (13.1 μg/ml ± 1.7, n = 20). Moreover, workers were also unaware of the effects of alcohol consumption on blood lead levels. Among other life style factors that possibly contribute to additional lead exposure in and outside the workplace, having meal at the work place was the prime candidate. About 88% of the respondents confessed that they had meal at the work place at least once in a day.

**Figure 5:**
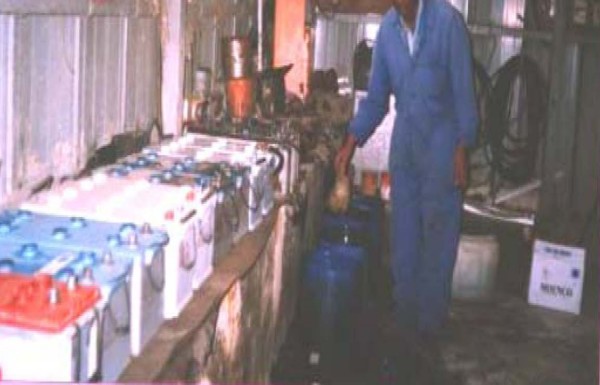
**A partial view of storage battery repair unit in one of the enterprises**. workers are engaged in routine activities without personal protective equipment, poor ventilation and full of dust.

**Figure 6:**
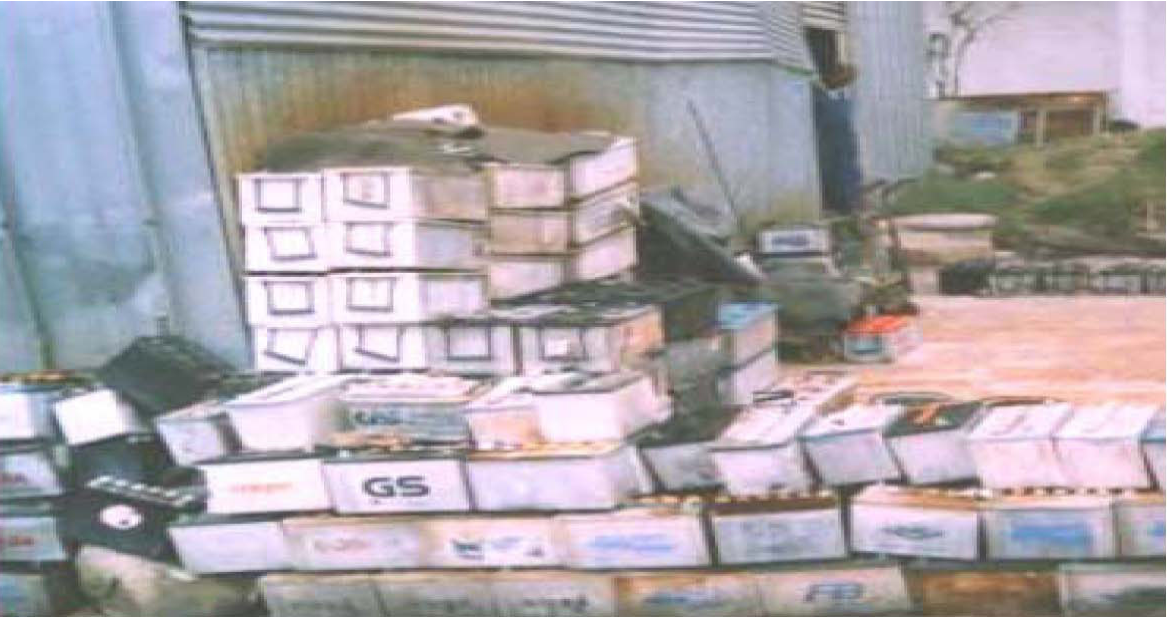
**Disposal of used storage batteries in one of the enterprises**. used batteries are not properly disposed and this has a long lasting environmental impact.

Interviews and walkthrough evaluation also revealed that none of the enterprises implement clear policy regarding the use of personal protective equipments (PPEs). All exposed subjects of the repair units reported that the enterprises had not provided training regarding lead toxicity. It was also observed during the walkthrough evaluation that all the enterprises workplace was dusty and did not follow lead regulations (Fig 5). Moreover, the way used batteries were disposed found to be inappropriate and hazardous to the environment (Fig 6), particularly to people living in the vicinity.

## Discussion

The application of biomarkers has become a crucial and widely used tool in understanding and assessment of health effects [1]. At present, blood lead levels are frequently measured to assess both lead exposure and effect that will facilitate the risk assessment process. However, a large body of evidence indicates that alternative biomarkers for lead that may be easily measured are also of major importance, particularly in the heme biosynthetic pathway [1,15]. Here we report for the first time the occupational hazard associated with lead exposure in Ethiopia.

### Urinary δ-ALA levels

This study considered urinary excretion of δ-ALA as a surrogate marker of blood lead in storage battery repair workers; owing to lack of facilities to measure blood lead levels. δ-ALA is excreted normally in small amounts in urine, but levels increase with lead exposure. Previous studies reported a five-fold increase in urinary excretion of δ-ALA following lead intoxication [16]. This rise in concentration of δ-ALA during lead exposure is a function of primarily decreased activity of enzymes involved in the heme synthetic pathway. This inhibition would then result in increased levels of δ-ALA in the blood and plasma, eventually leading to increased δ-ALA urinary excretion [15,17].

Increased urinary δ-ALA levels found in exposed subjects in the present study might be the impact of low-level long-term lead exposure at the repair units and reinforces the notion that δ-ALA can serve as a surrogate marker for lead exposure. In addition, the high urinary δ-ALA levels obtained from about 33.33% of exposed workers (Fig. 2) is a clear indicator of cumulative lead exposure and appears to be directly related to duration of employment at the repair units (Table 2). Evidence for the contribution of lead exposure to elevated urinary δ-ALA levels comes from the observation that 84% of non-exposed subjects exhibited normal range and none of them had high levels. This observation excludes the possibility that other factors might have contributed to the observed high levels of δ-ALA in exposed subjects. Chronic lead exposure as a culprit for higher δ-ALA levels was also corroborated by the observation that levels vary with duration of employment. Urinary δ-ALA levels in workers who had served for 25 years was about fourfold to those served for ten years and below. This finding is consistent with other reports that show urinary δ-ALA of lead workers increases with an increase in the duration of exposure [18].

Although findings published in the literature show that both age and gender have influence on blood lead levels [19], age but not sex was found to have effect on urinary δ-ALA in the present study. Sex was found to have little or no impact on urinary δ-ALA levels among the exposed subjects, though females are expected to have higher blood lead levels compared to males. This might have something to do with small number of females available for comparison. Whilst age was found not to be a necessary or sufficient factor for levels of urinary δ-ALA in non-exposed subjects, it had a significant correlation in exposed subjects. Plasma lead levels are known to be higher in children and decline with age, as bone density increases and lead starts to redistribute to the skeletal pool. However, in older people plasma lead again increases due to decalcification of bones and eventual release of lead into the plasma. Given this fact, the association of urinary δ-ALA with age could probably be better explained by duration of exposure rather than increase with age *per se*, as the maximum age of an exposed subject is an unlikely age where decalcification of bone starts.

Lifestyle factors other than the occupational settings can have an effect on the exposure of a toxicant. Such factors usually include smoking and alcohol taking. In this study, the effect of alcohol, particularly draught beer, on urinary δ-ALA levels of exposed subjects was analyzed and alcohol-taking subjects displayed increased levels than their non-alcohol-taking peers and this is in line with other reports [20]. The role of alcohol in blood lead levels is unclear and is still a subject of controversy. Published reports indicate that the draught dispensing equipment rather than alcohol *per se *is responsible for the increased lead concentration in alcohol-taking subjects [21]. They argue that the equipment sometimes contains brass or gunmetal that has low but significant amounts of lead. Thus, it is plausible to assume that the same argument might hold true for the observed increased urinary δ-ALA in alcohol-taking exposed subjects.

### Serum creatinine, creatinine clearance and urea levels

There is evidence to suggest that chronic low level lead exposure may affect kidney function [22,23]. However, the level of severity and duration of exposure leading to renal damage is not clearly defined. Though urinary δ-ALA increased in exposed subjects and appeared to be related to duration of employment, none of the renal indices were found to be different from the non-exposed subjects. Surprisingly, levels of serum creatinine, creatinine clearance and blood urea levels of both non-exposed and exposed subjects were found to be within the normal range (data not shown). Cross-sectional studies conducted in lead-exposed workers showed that lead might not cause adverse effects on renal glomerular and proximal tubular functions when there is long-term and less severe exposure [24,25]. Lack of renal effects in this study may point to the fact that exposure is not sufficient enough to bring about appreciable damage to the kidney. The notion that kidney damage is a function of degree/intensity of exposure is supported by other studies [26]. These authors found that exposed workers at the smelter had a greater serum creatinine levels and renal dysfunction, indicating that workers at the primary lead smelters have a higher chance of kidney damage than those in repair units.

### Uric acid levels

A relationship between gout and lead nephropathy has been recognized for centuries and gout occurs more frequently in the presence of chronic lead nephropathy than in any other type of chronic renal disease [22]. The fact that large proportion of exposed subjects had high serum uric acid levels than non-exposed ones is an indicator for the possible contribution of lead exposure (Fig. 4). Consistent with our finding, a growing body of evidence indicates that chronic occupational lead exposure is associated with low urate excretion [7,26]. Attempts were also made to examine additional factors other than lead exposure that might contribute for the rise in the levels of uric acid in both exposed and non-exposed subjects. And it was known that among exposed and non-exposed subjects there was no one who had been taking medication(s) that could contribute for the rise in the levels of uric acid, ruling thus out this possibility.

### Public health impact of the finding

In Ethiopia, there is no workplace regulation for lead exposure. Therefore, workers at lead acid battery units of the studied transport enterprises are clearly at high risk of lead exposure (Fig 5), as 39% of exposed workers had some of the common illnesses associated with lead poisoning. Not only the workers, but also people living nearby the repair units are at high risk of exposure due to failure to follow proper disposal method for used batteries (Fig 6). It was also interesting to note that 44% of exposed subjects reported that they had changed workstations through promotion but not because of the risks of lead exposure, which would definitely affect productivity of workers in the long run [10,27]. Health risks of lead require due attention by the enterprises management and periodic medical checkups should be put in place along with promoting awareness about the risks associated with lead exposure. It may not be feasible to quickly introduce engineering controls so as to protect storage battery repair workers. Biological monitoring from urine and/or blood samples would, however, be useful in identifying and lowering excess lead absorption. Furthermore, workers should use PPEs very strictly. Enterprises need a clear policy regarding proper use of PPEs [28], besides training and regular supervision of workers.

In another finding of this study, the structured questionnaire analysis showed that 88% of exposed subjects had meals at workplaces on regular basis for at least once per day and were assumed to have additional lead exposure. All the enterprises should explore the possibility of establishing cloth changing facilities, decontamination services, and dining rooms to ensure good performance and well being of workers [29]. Improvements of hygienic practices are more effective at lowering blood lead levels than reducing ambient lead level [30]. Hygienic practices might therefore be the preferred way to reduce lead exposure at the workplace, especially in developing countries like Ethiopia compared to the engineering controls.

Lead poisoning is a preventable disease provided an integrated prevention program is organized and maintained. Safety and health measures, such as general ventilation are usually desirable to control exposure to airborne substances by diluting the airborne contaminants [9]. Ethiopian lead regulations need to be developed and regular progress monitoring should be made in instituting new workplace lead controls, implementing large scale health screenings and lowering this all-pervasive and hidden epidemic, so that occupational lead exposure and its long-term impacts on society are ultimately eliminated.

To sum up, raised levels of urinary δ-ALA and uric acid obtained from the exposed subjects may indicate the possible parallel rise in blood lead levels. These measured values were mainly attributed from poor preventive and control measures at the repair units. Improving the work environment of the workers is quite important, as the next workers who are assigned to work in the 'non-fit' environment would also be exposed to the same hazard that entails an overall decrease in productivity of the enterprises. By and large occupational exposure to lead remains a big problem in developing countries including Ethiopia. Therefore, it is necessary that lead exposures at workplaces be minimized by placement of appropriate and cost-effective integrated preventive and control measures.

## Declaration of competing interests

The authors declare that they have no competing interests.

## Authors' contributions

KA conception and design of the work, generation and analysis of data, GA generation and data analysis and commented the MS, EE conception and design of the work, data analysis, drafted and developed the MS.

## References

[B1] International Program on Chemical Safety (1995). Environmental Health Criteria 165: Inorganic Lead. Geneva.

[B2] Massaro EJ (1997). Handbook of Human Toxicology.

[B3] Winder C, Neill H Stacey (1993). Toxicity of metals. Occupational Toxicology.

[B4] Wu TN, Shen CY, Ko KN, Guu CF, Gau HJ, Lai JS, Chen CJ, Chang PY (1996). Occupational Lead Exposure and Blood Pressure. Int J Epidemio.

[B5] Robert AG, Thomas WC, Curtis DK (2001). Toxic Effects of Metals. Casarett and Doull's Toxicology: The Basic Science of Poisons.

[B6] World Health Organization Regional Office for Europe (2001). Air Quality Guidelines. Copenhagen.

[B7] Lin JL, Tan DT, Ho HH, Yu CC (2002). Environmental lead exposure and urate excretion in the general population. Am J Med.

[B8] Goelzer B (1996). The harmonized development of occupational hygiene: A need in developing countries. Am Ind Hygiene Association J.

[B9] World Health Organization (1999). Hazard prevention and control in work environment: Airborne Dust Geneva.

[B10] George AM, (Ed) (1999). Proceedings of the International Conference on Lead Poisoning Prevention and Treatment: 8–10 February 1999; Bangalore.

[B11] Henry JB (1984). Clinical Diagnosis and Management by Laboratory Methods.

[B12] Labbe RF, Lamon JM, Tietz NW (1987). Porphyrins and Disorders of Porphyrins Metabolism. Fundamentals of Clinical Chemistry.

[B13] Tomokumi K, Ogata M (1972). Simple method for determination of urinary δ-ALA as an index of lead exposure. Clin Chem.

[B14] Lane RE, Hunter D, Malcolm D, Williams MK, Hudson AR, de Kretser AJ, Zielhuis RL, Cramer K, Barry PSI, Beritic T, Vigliani EC, Truhaut TR, Kehoe RA, King E (1968). Diagnosis of inorganic lead poisoning: A statement. Brit Med J.

[B15] Sakai T (2000). Biomarkers of Lead Exposure. Ind Health.

[B16] Bauer JD (1982). Clinical Laboratory Methods.

[B17] Higashikawa K, Furuki K, Takada S, Okamoto S, Ukai H, Yuasa T, Ikeda M (2000). Blood Lead Level to Induce Significant Increase in Urinary δ-Aminolevulinic Acid Level among Lead-Exposed Workers: A Statistical Approach. Industrial Health.

[B18] Lee BK (1982). Occupational lead exposure of storage battery workers in Korea. Br J Ind Med.

[B19] Hodgkings DG, Hinkamp DL, Robins TG, Schork MA, Krebs WH (1991). Influence of high past lead-in-air exposures on the lead – in – blood levels of lead – acid – battery workers with continuing exposure. J Occup Med.

[B20] Weyermann M, Brenner H (1997). Alcohol consumption and smoking habits as determinants of blood lead levels in a national population sample from Germany. Arch Environ Health.

[B21] Sherlock JC, Pickford CJ, White GF (1986). Lead in alcoholic beverages. Food Addit Contam.

[B22] Bernard BP, Becker CE (1988). Environmental Lead Exposure and the Kidney. J Toxicol Clin Toxicol.

[B23] Lim YC, Chia KS, Ong HY, Ng V, Chew YL (2001). Renal dysfunction in workers exposed to inorganic lead. Ann Acad Med Singapore.

[B24] Omae K, Sakurai H, Higashi T, Muto T, Ichikawa M, Sasaki N (1990). No adverse effects of lead on renal function in lead-exposed workers. Ind Health.

[B25] Wang VS, Lee MT, Chiou JY, Guu CF, Wu CC, Wu TN, Lai JS (2002). Relationship between blood lead levels and renal function in lead battery workers. Int Arch Occup Environ Health.

[B26] Pinto de Almeida AR, Carvalho FM, Spinola AG, Rocha H (1989). Renal dysfunction in Brazilian lead workers. Am J Nephrol.

[B27] Smith JM, Stellman JM (1997). Ergonomics factors. Occupational Health and safety Encyclopedia.

[B28] Stellman JM, Osinsky D (1997). Encyclopaedia of Occupational Health and Safety.

[B29] International Labour Office (1996). Ergonomics Checkpoints: Practical and easy to implement solutions for improving safety, health and working conditions. Geneva.

[B30] Lai JS, Wu TN, Liou SH, Shen CY, Guu CF, Ko KN, Chi HY, Chang PY (1997). A study of the relationship between ambient lead and blood lead among lead battery workers. Int Arch Occup Environ Health.

